# A Systems Biology Approach to Deciphering the Etiology of Steatosis Employing Patient-Derived Dermal Fibroblasts and iPS Cells

**DOI:** 10.3389/fphys.2012.00339

**Published:** 2012-09-03

**Authors:** Justyna Jozefczuk, Karl Kashofer, Ramesh Ummanni, Frauke Henjes, Samrina Rehman, Suzanne Geenen, Wasco Wruck, Christian Regenbrecht, Andriani Daskalaki, Christoph Wierling, Paola Turano, Ivano Bertini, Ulrike Korf, Kurt Zatloukal, Hans V. Westerhoff, Hans Lehrach, James Adjaye

**Affiliations:** ^1^Department of Vertebrate Genomics, Max Planck Institute for Molecular GeneticsBerlin, Germany; ^2^Institute of Pathology, Medical University of GrazGraz, Austria; ^3^Division of Molecular Genome Analysis, German Cancer Research CenterHeidelberg, Germany; ^4^Manchester Centre for Integrative Systems Biology, Manchester Interdisciplinary Biocentre, University of ManchesterManchester, UK; ^5^Cancer Stem Cell Group, Institute for Pathology and Comprehensive Cancer Center, Charité – UniversitätsmedizinBerlin, Germany; ^6^Magnetic Resonance Center, University of FlorenceFlorence, Italy; ^7^Department of Molecular Cell Biology, Netherlands Institute for Systems Biology, Vrije Universiteit AmsterdamAmsterdam, Netherlands; ^8^Doctoral Training Centre ISBML, The Manchester Centre for Integrative Systems Biology, Manchester Interdisciplinary Biocentre, University of ManchesterManchester, UK; ^9^Synthetic Systems Biology, Swammerdam Institute for Life Sciences, University of AmsterdamAmsterdam, Netherlands; ^10^Dahlem Centre for Genome Research and Medical Systems BiologyBerlin, Germany; ^11^Medical Faculty, Institute for Stem Cell Research and Regenerative Medicine, Heinrich Heine UniversityDüsseldorf, Germany

**Keywords:** NAFLD, induced pluripotent stem cells, sterol biosynthesis, glutathione metabolism, lipid metabolism, AKT/mTOR signaling, systems biology, modeling

## Abstract

Non-alcoholic fatty liver disease comprises a broad spectrum of disease states ranging from simple steatosis to non-alcoholic steatohepatitis. As a result of increases in the prevalences of obesity, insulin resistance, and hyperlipidemia, the number of people with hepatic steatosis continues to increase. Differences in susceptibility to steatohepatitis and its progression to cirrhosis have been attributed to a complex interplay of genetic and external factors all addressing the intracellular network. Increase in sugar or refined carbohydrate consumption results in an increase of insulin and insulin resistance that can lead to the accumulation of fat in the liver. Here we demonstrate how a multidisciplinary approach encompassing cellular reprogramming, transcriptomics, proteomics, metabolomics, modeling, network reconstruction, and data management can be employed to unveil the mechanisms underlying the progression of steatosis. Proteomics revealed reduced AKT/mTOR signaling in fibroblasts derived from steatosis patients and further establishes that the insulin-resistant phenotype is present not only in insulin-metabolizing central organs, e.g., the liver, but is also manifested in skin fibroblasts. Transcriptome data enabled the generation of a regulatory network based on the transcription factor SREBF1, linked to a metabolic network of glycerolipid, and fatty acid biosynthesis including the downstream transcriptional targets of SREBF1 which include LIPIN1 (LPIN) and low density lipoprotein receptor. Glutathione metabolism was among the pathways enriched in steatosis patients in comparison to healthy controls. By using a model of the glutathione pathway we predict a significant increase in the flux through glutathione synthesis as both gamma-glutamylcysteine synthetase and glutathione synthetase have an increased flux. We anticipate that a larger cohort of patients and matched controls will confirm our preliminary findings presented here.

## Introduction

Non-alcoholic fatty liver disease (NAFLD) is on the rise in the western population affecting one-third of adults and an increasing proportion of children (de Alwis and Day, 2008; Vuppalanchi and Chalasani, 2009). Early stages of this disease exhibit accumulation of lipids in the liver leading to hepatic steatosis which is followed by advancing levels of fibrosis and inflammation. In a subset of these individuals the inflammatory response is accompanied by hepatocyte ballooning and progression to steatohepatitis which can lead to cirrhosis and liver cancer (Day, 2006; Petta et al., 2009).

Hepatic steatosis is caused by the abnormal accumulation of predominantly triglycerides (TG) in the liver. In histological sections of liver parenchyma TG droplets are visible in the cytoplasm of hepatocytes. This accumulation of TG in small or large vesicles impairs hepatic function and makes the liver more susceptible to other injuries. It has been shown that patients with NAFLD have a worse outcome when compared with an age and sex-matched general population, stemming from cardiovascular and liver related mortality (Ekstedt et al., 2006; Angulo, 2010). As most patients with NAFLD are obese and often resistant to insulin, steatosis can lead to significant co-morbidity or progress to non-alcoholic steatohepatitis, thus justifying therapeutic intervention already at the early stages. As NAFLD is highly associated with obesity and diabetes, weight loss can greatly improve the health status of these patients. However, the different susceptibility of patients with similar body mass index (BMI) to acquire NAFLD suggests a role for genetic factors and susceptibility genes.

The liver utilizes glycogen for energy storage and TG are usually mainly stored in adipocytes. Fatty acids originate either from the circulation, i.e., diet, are released from adipocytes by adipocyte TG hydrolase (ATGL) or are synthesized *de novo* in the liver. In each of these metabolic pathways, multiple transcription factors and signaling cascades (Horton et al., 2002; Li et al., 2010b) are involved thus presenting multiple possibilities for regulation and also deregulation of this network.

There exist several hereditary genetic disorders that demonstrate that these networks can be deregulated *in vivo*. Congenital generalized lipodystrophy, glycogen storage disease type 1a, and citrin deficiency all cause severe hepatic steatosis even in the absence of obesity or insulin resistance. Several mutations and genetic defects are known to influence TG accumulation in the liver, like mutations of ATGL which prevent mobilization of TG from the hepatocytes or mutations in apolipoprotein B which impair TG secretion to the bloodstream (Hooper et al., 2011). However, single gene mutations are only rarely responsible for NAFLD with the majority of cases caused by changes in the quantity and composition of food consumed in the western hemisphere. The Dallas Heart Study, which included 2000 individuals of multi ethnic origin, has shown that hepatic steatosis is rare in lean individuals with a BMI < 25 kg/m^2^ whereas it is highly prevalent in obese participants presenting a BMI > 45 kg/m^2^ (Browning et al., 2004).

In summary, hepatic steatosis is clearly correlated to food intake and insulin resistance. However, even in the severely obese, large differences in the extent of steatosis can be observed. As steatosis stands at the start of the road to non-alcoholic steatohepatitis (NASH) and hepatocellular carcinoma (HCC), finding genetic determinants of susceptibility to NAFLD might help in the prevention of NASH and HCC.

Studying NAFLD in human patients is limited by the need for liver biopsies to determine the stage of NAFLD and by the strong influence of the individual environment of the patient with respect to food intake and exercise. Nevertheless some genome wide association studies have recently been performed and identified genetic variants that have distinct effect on metabolic traits (Speliotes et al., 2011). Animal models have shed light on a causal relationship between insulin resistance and hepatic steatosis but so far failed in elucidating useful genetic markers to determine the susceptibility of individuals to develop NAFLD (Voshol et al., 2003; den Boer et al., 2004).

Hepatocytes generated from induced Pluripotent Stem (iPS) cells can thus present a model system in which the response of naïve hepatocytes with the patient’s genome to experimental stimuli can be tested and analyzed. These unique features of iPS cell-derived hepatocytes could help in elucidating the genetic networks that are responsible for the different reaction of the patients hepatocytes to the environmental stimulus presented to them by the patient’s lifestyle.

**Figure 1:**
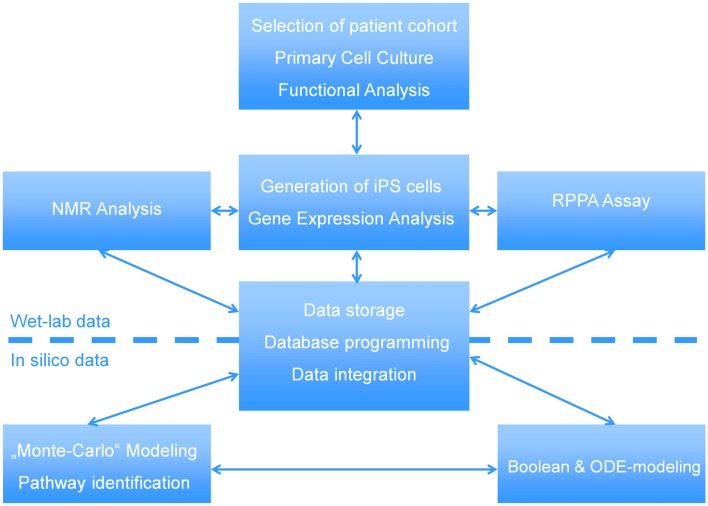
**Interdependencies between the wet-lab data and *in silico* data to decipher the biology of NAFLD**. The complex systems biology of NAFLD is explored by a multidisciplinary consortium of clinical and research groups having profound expertise in the fields of stem cells, pathology of chronic liver and metabolic disorders, development of a Biobank to support drug discovery, high-throughput sequencing, functional genomics, bioinformatics and systems biology. ODE-modeling refers to Ordinary Differential Equation-based modeling.

In this manuscript we illustrate how a multi-disciplined systems biology approach can be used to study the etiology of steatosis. The scheme presented in Figure 1 shows the interactions between wet-labs (cell biology and biochemistry) and *in silico* analysis which is made up of data integration, pathway reconstruction, and modeling. A description of the consortium can be found at http://www.erasysbio.net/index.php?index=264.

## Materials and Methods

### Patient recruitment and sample collection

Starting in 2009, patients with morbid obesity were offered to participate in the Multidisciplinary Obesity Research (MORE) project at the Medical University of Graz, Austria. This study aims to take an interdisciplinary approach in research on obesity and related diseases like insulin resistance, liver steatosis and metabolic syndrome and includes the Institutes of Gastroenterology, Pathology, Surgery and Experimental Hepatology at the University Hospital of Graz. Patients with morbid obesity admitted into hospital for treatment by bariatric surgery (gastric banding, gastric bypass, sleeve gastrectomy) were invited to participate in the study and to sign the informed consent. In addition to obese patients we also included three healthy individuals in the study to obtain control dermal fibroblasts. If patients were admitted for bariatric surgery, samples of blood, skin and a liver biopsy were taken during the operation. The skin samples were immediately transported to the Institute of Pathology and fibroblast cultures were initiated. The liver biopsy was divided into one part for fixation and histology and a second part for liquid nitrogen storage while the blood sample was separated into mononuclear cells and plasma. Subsequently the disease phenotype characterization comprised detailed analysis of glucose (e.g., type 2 diabetes, insulin resistance) and lipid metabolism, hypertension, atherosclerosis, and histopathological evaluation of the liver biopsy. In addition, HOMA IR (homeostatic model assessment insulin resistance) was calculated for patients. The average normal-weight individual has an insulin resistance of 1, while for obese patients recruited for the study the HOMA IR index is within the range from 2.82 to 4.86, indicating the insulin resistance phenotype. Moreover, other diseases had to be excluded, and a detailed evaluation of the lifestyle (nutrition, physical activity and consumption of alcohol or drugs) was performed from the structured questionnaires and, where applicable, biomarkers and physical measurements.

### Derivation of dermal fibroblast cells from patients

Primary fibroblasts were derived by standard tissue culture practice. In brief, the skin surface was sterilized with ethanol and subcutaneous fat was removed. The sample was divided into small pieces (2 mm × 2 mm) and each of these pieces was minced in a culture dish, supplemented with 1 mL of DMEM, 10% FCS with antibiotics, and covered with a sterile coverslip. After approximately 2 weeks skin fibroblasts started to grow out from the skin pieces and after approximately 4 weeks the fibroblasts were harvested and expanded.

Primary fibroblasts from two patients with steatosis (H0007, H0008) and a non-obese control (H0002) were established.

### Protein lysate preparation

After washing with PBS two times cells were directly lysed in M-PER buffer (Pierce) with EDTA free complete inhibitors (Roche) and phosphostop (Roche). Lysates were immediately frozen at −80°C. Further samples were allowed to thaw on ice and incubated on a rotating shaker at 4°C for 20 min. To clear protein samples, lysates were centrifuged at maximum speed for 30 min at 4°C. The protein supernatant was collected and protein concentration was estimated using a micro BCA method (Smith et al., 1985).

### Reverse phase protein array

The method used in this study has been described previously (Loebke et al., 2007). For each sample four replicate spots were printed using the 2470 Arrayer (Aushon Biosystems, Billerica, USA). Slides not used immediately were kept a −20°C and rehydrated in PBS-Tween (0.05%). Blocking was carried out using Odyssey Blocking buffer (LI-COR, Lincoln, USA) diluted to 1:1 with PBS-Tween (0.05%) containing NaF and NaVO_3_ for 1h at room temperature. Next, slides were incubated with pre-validated antibodies (Table 1) at 1:300 dilutions in blocking buffer overnight at 4°C. Primary antibody detection was carried out with Alexa-680 labeled goat-anti-rabbit or goat-anti-mouse secondary antibodies and visualized using an Odyssey scanner (LI-COR, Lincoln, USA). Array images were analyzed and quantified using GenePix Pro 5.0 software (Molecular Devices, Ismaning, Germany). For on-slide protein quantification the total protein content of single spots was determined according to the FAST Green-FCF assay (Loebke et al., 2007).

**Table 1:** **List of the pre-validated antibodies used in reverse phase protein array (RPPA)**.

Target	Phosphosite	Antibody ID	Company
ERK1/2	T202Y204	4370	Cell Signaling Technology
PRAS40	T246	2997	Cell Signaling Technology
AKT	S473	4058	Cell Signaling Technology
PEPCK		sc-32879	Santa Cruz Biotechnology
4EBP1		NB200157	Novus Biologicals
TSC1		4906	Cell Signaling Technology
TSC2		3990	Cell Signaling Technology
CD36		AF2519	R&D Systems

### Data analysis of reverse phase protein arrays

Data analysis was performed using the software tool RPPanalyzer (Mannsperger et al., 2010) implemented in the statistical environment R. Arrays with signal intensities not significantly above blank readings as well as arrays without a linear correlation between signal intensity and protein concentration were not analyzed. Significance was calculated applying two-sample t-tests. Raw data was normalized for total protein content using FCF data as described before (Loebke et al., 2007). To obtain significantly regulated proteins, t-test statistics were applied. Three biological replicates and five technical replicates were tested and *p*-value was adjusted for multiple testing.

### Transcriptome analysis of patient specific fibroblasts and control

Gene expression analysis was performed using the Illumina HumanRef-8 v3 BeadChip. The bead-level data generated by the image scanner software were summarized and saved without background correction and normalization in the Illumina BeadStudio. Bead-summary data was analyzed via R/Bioconductor (Gentleman et al., 2004) and the lumi package (Du et al., 2008) starting with background subtraction, variance-stabilizing transformation and quantile normalization. Fibroblasts from two steatotic patients (H0007, H0008) were compared to control fibroblasts from a non-steatotic person (H0002). For each patient two replicates were hybridized. Differential expression was tested using Welch’s test and the moderated t-test from the limma (Smyth, 2004) package test in parallel. The list of significantly expressed genes was based on limmap, which represents the *p*-value from moderated t-test from limma-Package (limmap < 0.05) and the *p*-value from Welch’s test (t2p < 0.05). The Bioconductor package *q*-value (Storey, 2002) was employed to control the false discovery rate. Genes were considered differentially expressed when the Illumina detection *p*-value (judging significance of discrimination from the microarray image background) of at least one of the control or treatment group and the false discovery rate were usually 0.05.

Microarray data analysis was then performed on data grouped into the following combinations: steatosis patient H0007 vs. control, steatosis patient H0008 vs. control and steatosis patients H0007 and H0008 vs. control in order to identify the major markers and molecular read-outs as well as annotation of the functional networks that interconnect these markers. All original gene array files are available from the Gene Expression Omnibus (GEO) database^1^ accession number GSE37689.

### Discovery of pathways and functional modules through network analysis for the development of computational models

Gene-set enrichment and over-representation analysis (ORA) was achieved utilizing the unique pathway database integration system ConsensusPathDB (Kamburov et al., 2009, 2011b) which is designed with a focus on human pathway and interaction data and integrates the content of most important pathway databases such as Reactome (Matthews et al., 2009; Croft et al., 2011), KEGG (Kanehisa et al., 2010), IntAct (Kerrien et al., 2007), DIP (Salwinski et al., 2004) as well as manually curated data resulting from experimental investigation or interaction data available in common pathway and systems biology file formats, such as SBML (Hucka et al., 2003), BioPAX (Demir et al., 2010), or PSI-MI (Hermjakob et al., 2004). The current content of the integration tool comprises more than 51,000 physical entities with more than 1,69,000 functional interactions. Pathway analysis with a daughter tool of ConsensusPathDB, IMPaLA (Kamburov et al., 2011a) has been used to identify pathways related to signaling and metabolism. In addition to over-represented pathways, also nearest neighborhood-based entity sets (NESTs) can be identified with ConsensusPathDB. NESTs are centered at a specific entity (gene, metabolite, etc.) and contain all direct interactions and interaction partners. Thus, NESTs can be viewed as unbiased (non-annotated) functional modules derived from the integrated network that can be used as gene sets for gene enrichment analyses as an alternative to curated pathways. NESTs can further be restricted with statistical graph-theoretical arguments, for example minimal connectivity, minimal degree, etc. Identified pathways and functional modules have been integrated in a prototype model related to disease development of steatosis and steatohepatitis.

### Modeling glutathione synthesis and metabolism pathways

For this work an updated version of the glutathione model previously published was used (Reed et al., 2008; Geenen et al., 2012). The model is available for simulation at all three JWS Online (Olivier and Snoep, 2004) servers (e.g., http://jjj.mib.ac.uk/webMathematica/Examples/run.jsp?modelName=geenen). The modified model consists of the methionine cycle, glutathione synthesis and detoxification, ophthalmic acid synthesis, and the gamma-glutamyl cycle. We are more confident in the ability of this model to make quantitatively accurate predictions as this model was fitting on more experimental data (than the previous model) and is partially validated (Geenen et al., manuscript in preparation). However we have not validated the model for predicting the effect in changes in *V*_max_ and therefore the predictions made here will offer limited certainty.

This model represents the control data set discussed in this study. The difference in expression between control (H0002) and steatosis (H0007, H0008) was first calculated and then used to calculate the *V*_max_ for H0007 and H0008. In order to take account of the differences of expression within the repeats, the *V*_max_ of all the repeats was calculated. This was then inputted into the model as a range of *V*_max_. The range of *V*_max_ inputted into the model for the control model (H0002), patient H0007, and H0008 are presented in Table A1 in Appendix.

Similarly, a range of *V*_max_s was also incorporated in the control model based on the range of expression data of the control patient. Three models were then analyzed: the control (H0002) model, H0007, and H0008. In order to calculate the fluxes/concentrations of the model, the model selected a *V*_max_ value for each enzyme within the new calculated range and the steady state was calculated. This was repeated 5,00,000 times using Condor-COPASI^2^ (Hoops et al., 2006). This resulted in 5,00,000 different steady states due to the different combinations of *V*_max_ values randomly chosen by COPASI. The average and standard deviation (SD) of flux/concentration was calculated from this data.

## Results

### Pathomorphological assessment of patient’s phenotype

**Figure 2:**
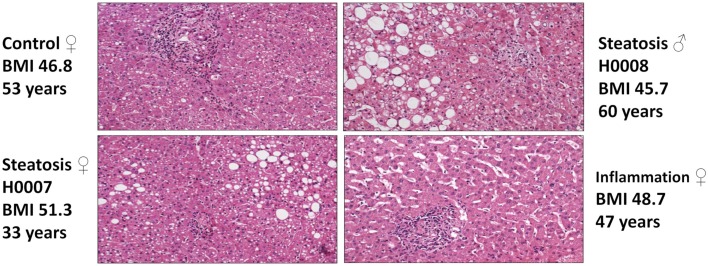
**Hematoxylin-Eosin stained sections of liver biopsies from the four patients enrolled in the study (top: control (left), steatosis (H0008, right); bottom: steatosis (H0007, left), sinusoidal dilatation and inflammation (right)**.

**Figure 3:**
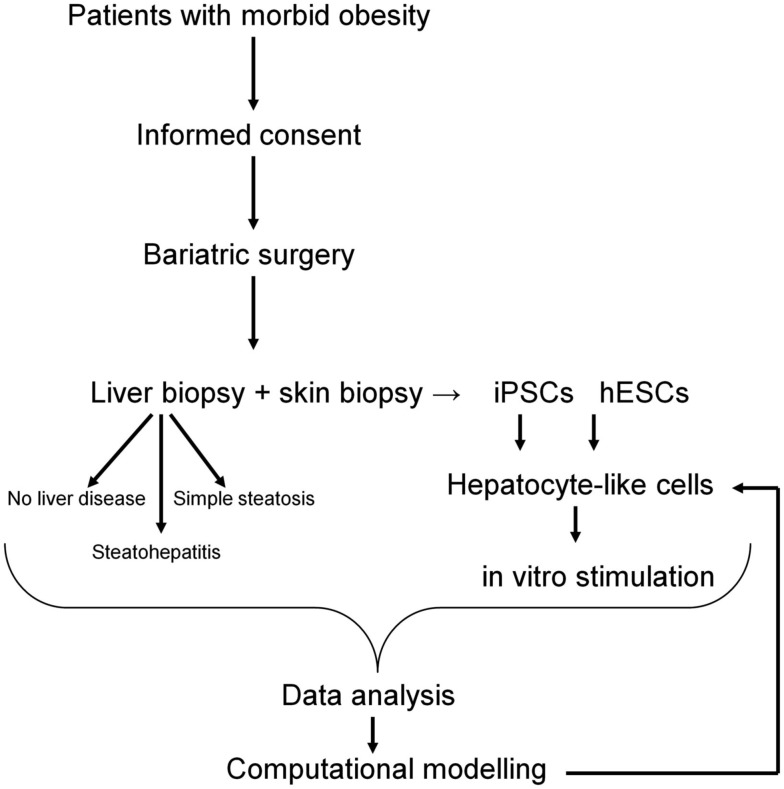
**Workflow of patients recruitment, sample collection, and downstream analyses**. The computational model of NAFLD will be generated based on hepatocyte-like cells challenged with “environmental” conditions typical for NAFLD. Data obtained from *in vitro* studies will be compared with data from liver biopsies and further validated *in vitro* on hepatocyte-like cells.

Pathomorphological assessment of the histology of the liver biopsies (Figure 2) allowed to select three patients with high grade steatosis (>30% steatosis, BMI 44 ± 3) and three patients with low grade steatosis (0–10% steatosis, BMI 42 ± 7) with similar BMI. These samples are supplemented by three control samples from healthy individuals. The number of samples is still small so sample acquisition is ongoing. The scheme in Figure 3 depicts the overall strategy for accomplishing our aims.

### iPS cell-based derivation of an *in vitro* disease model of steatosis

**Figure 4:**
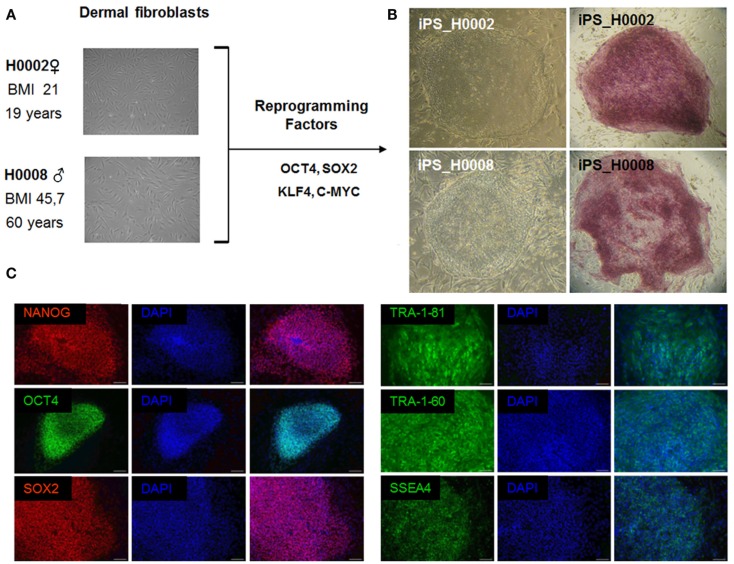
**The patient-specific fibroblasts were transduced with the ES cell specific transcription factors OCT4, SOX2, KLF4, and c-MYC**. Then grown on MEFs in ES cell culture medium. After 3 weeks flat ES-like colonies were observed and picked mechanically. **(A)** Phase contrast images of dermal fibroblasts derived from skin biopsies of steatosis patient (H0008) and control individual (H0002). **(B)** Phase contrast images of iPS cells derived from dermal fibroblasts and staining of alkaline phosphatase which is a marker associated with undifferentiated pluripotent stem cells. **(C)** Characterization of iPS cells. iPS cells express the crucial pluripotency-associated transcription factors NANOG, OCT4, SOX2 as well as cell surface markers TRA-1-81, TRA-1-60, and SSEA4. Scale bar = 100 μm.

Retroviruses encoding the reprogramming factors, OCT4, SOX2, KLF4, and c-MYC were transduced into fibroblasts cells from patient with steatosis (H0008) and control (H0002) using well established protocols (Takahashi et al., 2007; Wolfrum et al., 2010). These iPS lines express the embryonic stem cell specific markers, NANOG, SOX2, OCT4, SSEA4, TRA-1-60, and TRA-1-81 (Figure 4).

### Comparative transcriptional analysis of dermal fibroblasts derived from steatotic patients (H0007 and H0008) and healthy individual serving as control (H0002).

**Figure 5:**
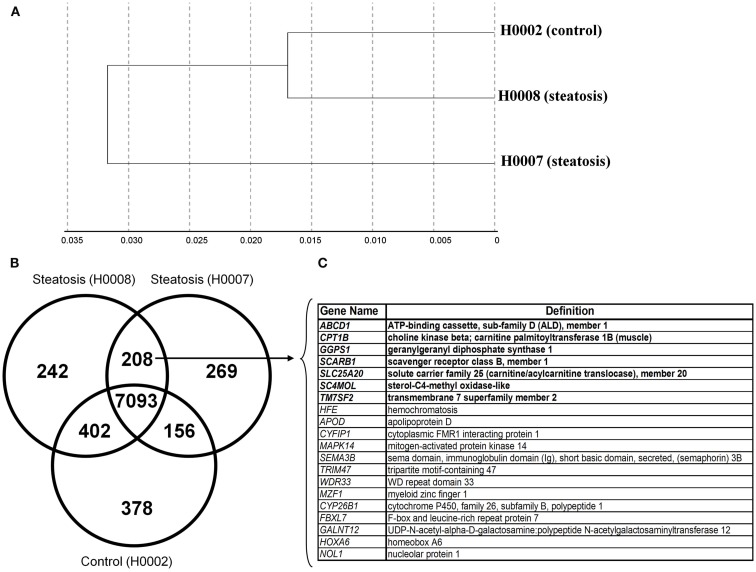
**Comparative transcriptional analysis of dermal fibroblasts from steatotic patients (H0007 and H0008) and healthy individual (H0002)**. **(A)** The clustering of respective transcriptomes. **(B)** The Venn diagram illustrating the distinct and overlapping genes. **(C)** List of 20 genes expressed in both patient-derived fibroblasts. Seven genes involved in metabolism of lipids and lipoproteins (highlighted in bold).

The transcriptomes of dermal fibroblasts derived from two patients with steatosis [60 year, male BMI 45.7 (H0008) and 33 year, female, BMI 51.3 (H0007)] and control fibroblasts [19 year, female, BMI 21 (H0002)] were analyzed. The clustering of the respective transcriptomes is shown in Figure 5A, the correlation co-efficient ranged from 0.92 to 0.96.

First we considered all genes expressed (detection *p*-value < 0.01) in steatotic (H0007, H0008) and control (H0002) fibroblasts. The Venn diagram in Figure 5B illustrates that there are 208 genes exclusively expressed in fibroblasts from patients with steatosis. Several examples of overlapping genes (including seven genes involved in metabolism of lipids and lipoproteins, highlighted in bold) are presented in Figure 5C. The complete list of 208 overlapping genes is provided in Table 2, amongst this list are genes (represented as CORFs, FLJs, and LOCs) of unknown function and therefore considered as novel steatosis-associated genes.

**Table 2:** **List of 208 genes expressed in common between fibroblasts derived from two steatotic patients**.

**GENES EXPRESSED IN COMMON BETWEEN FIBROBLASTS FROM STEATOTIC PATIENTS (H0007 and H0008)**							
ABCD1	C9ORF111	DLX1	HCAP-H2	MGAT2	PI16	SEMA3B	TRAPPC6A
ACSS1	C9ORF150	DMWD	HFE	MGC13024	PIGZ	SEMA5A	TREX1
AKAP12	C9ORF16	DOCK6	HIST1H4H	MMP11	PITX1	SGK3	TRIM16L
ALKBH1	CART1	DSCAM	HOXA6	MORC4	PLAGL1	SLC12A8	TRIM47
AMPH	CASP9	DYRK1B	HOXC10	MRPL10	POMZP3	SLC1A3	TRIM9
ANK3	CCNL2	DYRK2	HTATIP	MTG1	PPP2R3B	SLC25A20	TTLL1
AP3M1	CCNT2	EDG4	IL17RB	MTHFR	PRICKLE1	SLC26A6	UBOX5
APOD	CD9	EFTUD1	IRX1	MX2	PRR3	SLC35C2	UNC13B
ARHGEF10L	CDKN2C	EPC1	KBTBD3	MYOM2	PSMF1	SLC7A7	USH1G
ASCC3	CDO1	F25965	KIAA1967	NBPF1	PSRC2	SLC9A9	UTP15
ATP5G2	CEP250	FAM102A	KIFC2	NFKBIZ	PTGDS	SLITRK4	VIT
BCR	CFD	FBXL7	KLF8	NGDN	PTP4A3	SNURF	VPS13C
BRWD2	CLYBL	FBXO3	LARP2	NME2	PTPN9	SNX1	VPS8
BTN3A3	CMYA5	FLJ13639	LGP2	NOL1	PTPRE	STAP2	VWCE
BUB3	CPT1B	FLJ20422	LMAN1	NTN4	PTX3	SYVN1	WDR33
BVES	CPZ	FLJ22167	LOC158160	OR11L1	RAB23	TACSTD2	WDR81
C11ORF11	CSPP1	FLJ40142	LOC652968	OSR2	RASIP1	TAPBP	WNT2
C14ORF28	CTSH	FLT3LG	LOC653626	PARP16	RBBP9	TBC1D10A	ZCRB1
C14ORF37	CXORF56	G3BP2	LRIG3	PARP6	RBMS3	TGIF2	ZFYVE27
C16ORF28	CYFIP1	GALNT12	LSM14B	PCDHB2	RDH13	THBS3	ZNF266
C1ORF115	CYP26B1	GALNTL1	MAFG	PCDHGB6	REEP6	TLE1	ZNF42
C20ORF161	DACT1	GALNTL2	MAPK14	PCNXL2	RGN	TLR4	ZNF521
C21ORF56	DAPK2	GEM	MBP	PDCD8	RNF8	TM4SF1	ZNF580
C21ORF63	DCTN1	GGPS1	MCPH1	PFKL	RRN3	TM7SF2	ZNF586
C5ORF4	DENND2A	GPR37	MDK	PHACS	SC4MOL	TP53INP1	ZNF589
C6ORF48	DHRS10	GUSBL2	MFI2	PHF11	SCARB1	TRAF3IP2	ZNFN1A2

### Target and pathway identification, annotation and computational modeling

**Figure 6:**
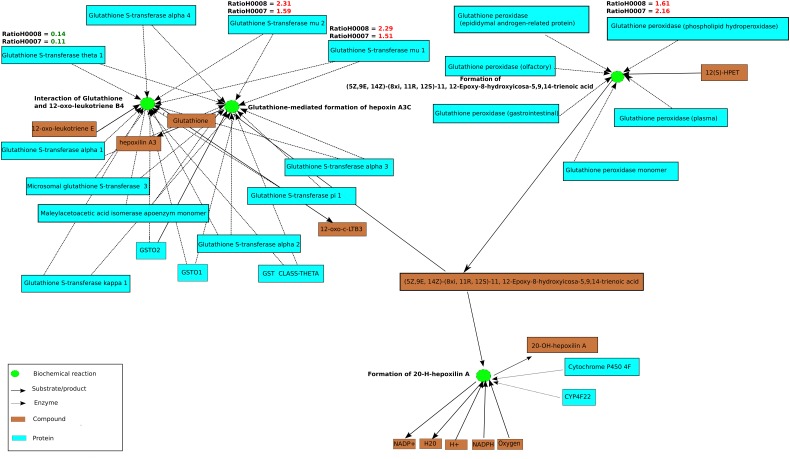
**A functional interaction sub-network visualized by ConsensusPathDB’s network visualization framework**. This sub-network defines the neighborhood-based entity set of genes playing a role in glutathione metabolism centered by cytochrome P450. The sub-network consists of 25 biochemical interactions (green circles), compounds, and metabolites are illustrated as light brown rectangles. Beyond the proteins that are visualized as light blue rectangles are the enzyme encoding genes *GSTT1*, *GSTM2*, *GSTM1*, and *GPX4* which are differentially regulated in patients H0007 and H0008 in comparison to the control, H0002. Ratios of *GPX4*, *GSTM2*, *GSTT1*, *GSTM1*, and *GSTA4* are also highlighted in red (up-regulation) or green (down-regulation). Four interactions from the KEGG Database are illustrated in this figure.

In order to assess whether the differentially expressed genes in steatosis patients (H0007 and H0008) compared to control (H0002) are enriched with respect to specific pathways, ORA was carried out with ConsensusPathDB. Using the hypergeometric test statistic of ConsensusPathDB on genes that are differentially expressed in the patients we were able to assign each pathway a *p*-value reflecting the occurrence of candidate genes for steatosis. ORA revealed top ranked pathways related to DNA replication (patient H0007, *p*-value = 3.22e−8; patient H0008, *p*-value = 2.95e−6) and cell cycle (patient H0007, *p*-value = 1.05e−6; patient H0008, *p*-value = 3.22e−8). Furthermore, ORA suggests that members of the NEST set related to glutathione metabolism (patient H0007, *p*-value = 0.0127; patient H0008, *p*-value = 5.61e−5) show jointly significant increase in transcription levels in steatosis patients. The NEST analysis is based on the genes glutathione S-transferase A4 (*GSTA4*), glutathione S-transferase theta-1 (*GSTT1*), and glutathione S-transferase mu 1 and 2 (*GSTM1*, *GSTM1*), which were identified as parts of the glutathione metabolism pathway, and the analysis identified a NEST gene-set that is centered around cytochrome *CYP4F22* (*p*-value = 8.67e−11, Figure 6). The gene *CYP4F22*, encodes a member of the cytochrome P450 superfamily of enzymes. Cytochrome P450 proteins are mono-oxygenases which catalyze many reactions involved in drug metabolism and synthesis of cholesterol, steroids, and lipids. The gene *GSTA4*, which is found to be up-regulated in the fibroblasts of the two steatosis patients, catalyzes the reaction of reduced glutathione to 4-hydroxynonenal, a product which is considered as a marker for oxidative damage in tissue. Furthermore, *GSTM1* and *GSTM2* that are found to be up-regulated in the fibroblasts of the two steatosis patients are known to be induced by oxidative stress. Moreover, glutathione peroxidase 4 (*GPX4*) is up-regulated in the steatosis patients and catalyzes the formation of the oxidized form of glutathione (glutathione disulfide, GSSG) from glutathione (GSH). GPX4 is the only antioxidant enzyme known to directly reduce phospholipid hydroperoxides within membranes and lipoproteins, acting in conjunction with alpha-tocopherol to inhibit lipid peroxidation (Sneddon et al., 2003). In contrast to *GSTM2*, *GSTM1*, *GSTA4*, and *GPX4* which are up-regulated in the steatosis patients, *GSTT1* is found to be down-regulated. *GSTT1* is known to be transcriptionally regulated by C/EBP-alpha which is considered to be critical for glucose and lipid homeostasis in adult mice (Inoue et al., 2004).

### Free fatty acids induced *de novo* lipid synthesis by activation of nuclear receptors SREBF1 and PPARG

Figure 11 depicts a minimal model of steatosis based on the patients transcriptome and associated pathways. The genes *LPIN* and *LDLR* which encode lipin and low density lipoprotein receptor (LDLR), respectively, are downstream targets of *SREBF1* (Fajas et al., 1999) and are up-regulated in both steatosis patients. *SREBF1* encodes a transcription factor that binds to the sterol regulatory element-1 (SRE1), which is a decamer flanking, e.g., the LDL receptor gene (*LDLR*) and other genes involved in sterol biosynthesis. In addition to the transcriptional activation of *LPIN* and *LDLR*, Fajas et al. reported that SREBF1 regulates the expression of PPARG during adipocytes differentiation. Ishimoto et al. (2009) demonstrated that the transcription of LPIN1 is mediated by SREBF1 and nuclear factor Y (NF-Y) and has been identified to be regulated by cellular sterols, which play an important role in lipid metabolism. LPIN1 is involved in glycerolipid biosynthesis and catalyzes the synthesis of diacylglycerol (DAG).

In addition, SREBF1 regulates the expression of the gene *PPARG* that encodes the peroxisome proliferator-activated receptor gamma which is a regulator of adipocyte differentiation. PPARG has been implicated in the pathology of numerous diseases including obesity, diabetes, atherosclerosis, and cancer. PPARs can form heterodimers with retinoid X receptors (RXRs) regulating the transcription of genes encoding lipid droplet (LD) proteins such as *PLIN1*, *PLIN2*, *PLIN3*, *PLIN5*, *FSP27*, and *HIG2*, known to be expressed in steatotic liver. LD proteins play a role in the pathophysiology of fatty liver disease where hepatocytes enriched with LD proteins contain vast amounts of lipids.

### Targeted proteomics using reverse phase protein arrays

Reverse phase protein arrays (RPPA) were used to analyze protein extracts derived from skin fibroblast cell lines obtained from two steatosis patients (H0007, H0008) and from a healthy lean control (H0002). In detail, the expression level of 109 proteins and phosphoproteins was quantified and several target proteins as well as phosphoproteins were identified as differentially regulated. For example, phosphorylation levels of major signaling proteins such as AKT, PRAS40, p70S6K (T389), ERK1/2 were strongly and significantly reduced in both patient-derived fibroblast cell lines whereas expression and phosphorylation of insulin receptor beta (IRβ) and its key adaptor protein IRS1 were comparable in control cells.

In animal models of diabetes as well as in human type 2 diabetes, a down-regulation of AKT/mTOR signaling indicates impaired insulin signaling (Sesti et al., 2001). Likewise, reduced p70S6K1 (T389) phosphorylation in response to insulin was previously reported as a characteristic feature of adipocytes obtained from patients with type 2 diabetes (Ost et al., 2010). This suggests that at the level of AKT/mTOR signaling, fibroblast cells obtained from steatosis patients phenocopy features of insulin resistance.

**Figure 7:**
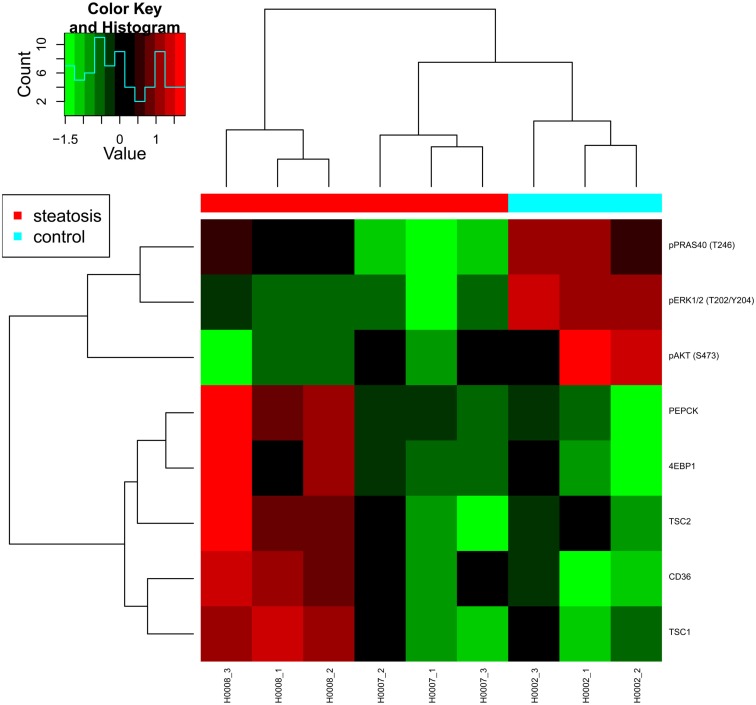
**Heat map presenting differentially expressed proteins**. Proteomic profiling was carried out for samples provided by the consortium such as fibroblasts from two patients with steatosis (H0007, H0008) and a non-obese control (H0002).

However, while animal models of insulin resistance suggest increased ERK signaling as well as an increased phosphorylation of IRS1 on its key activation site S632 (corresponding to S636 in human IRS1; Bouzakri et al., 2003), a significant down-regulation of phosphorylated ERK was observed for both patient-derived fibroblast cell lines. Patient H0008 cells revealed also decreased levels of IRS S636 (Figure 7). Thus, at the level of ERK signaling, notable differences exist between features characteristic for animal models of diabetes and fibroblast cells derived from steatosis patients.

The fatty acid translocase CD36 was up-regulated at the protein level in both steatosis patient samples although only significantly in cells derived from patient H0008, thus suggesting an increased uptake of lipids in steatosis patients. An up-regulation of CD36 in liver cells has also been associated with insulin resistance (Miquilena-Colina et al., 2011). Furthermore, fibroblasts from patient H0008 showed elevated expression of phosphoenolpyruvate carboxykinase 2 (PEPCK), a major enzyme of gluconeogenesis. Up-regulation of PEPCK and CD36 was previously reported to occur after inhibition of mTOR signaling by rapamycin (Houde et al., 2010) which is mimicked by the up-regulation of mTOR inhibitor proteins TSC1 and TSC2 in fibroblast cells derived from patient H0008. In conclusion, our data indicate impaired AKT/mTOR signaling exists in fibroblast cells derived from steatosis patients and that numerous features typical for diabetes type 2 were also observed in steatosis patients. Thus, molecular features of insulin resistance are present in skin fibroblasts of steatosis patients.

However, the question how other signaling pathways such as those mediated by ERK and PKCα contribute to the insulin-resistant phenotype and how this impacts on phosphorylation of IRS1 as key regulator of insulin responsiveness remains to be elucidated. Targeted proteomics carried out on larger numbers of human steatosis samples will certainly provide a meaningful contribution to the ongoing debate on molecular mechanisms that are causative for insulin resistance and thus also identify points for clinical intervention contributing to personalized therapies of steatosis patients.

### Modeling the glutathione synthesis pathways in dermal fibroblasts from steatosis and control

Preliminary investigation involved analysis of the transcriptome profiles, Gene Ontology (GO) ORA and KEGG pathway analysis, which selected for the implementation of a kinetic model for glutathione synthesis and metabolism (updated from the previous model published by Geenen et al., 2012). In H0007 vs. control (H0002), enrichment of filtered gene lists was observed in the following pathways: glutathione metabolism, metabolism of xenobiotics by cytochrome P450, and specifically drug metabolism by cytochrome P450. Similarly, in H0008 vs. control (H0002), enrichment of filtered genes was observed in the aforementioned pathways but also the TGF-β signaling pathway. Combining data sets from both patients selected for the glutathione synthesis and metabolism pathways to be of interest, forming the basis for the construction/implementation of a kinetic model.

### Glutathione pathway

Our hypothesis was that the flux through the glutathione pathway of the steatosis patients would be higher due to the steatosis grade (as determined from histology). We acknowledge clinical projects are often challenged with unbiased sample numbers, so we compared each patient data with control independently.

**Figure 8:**
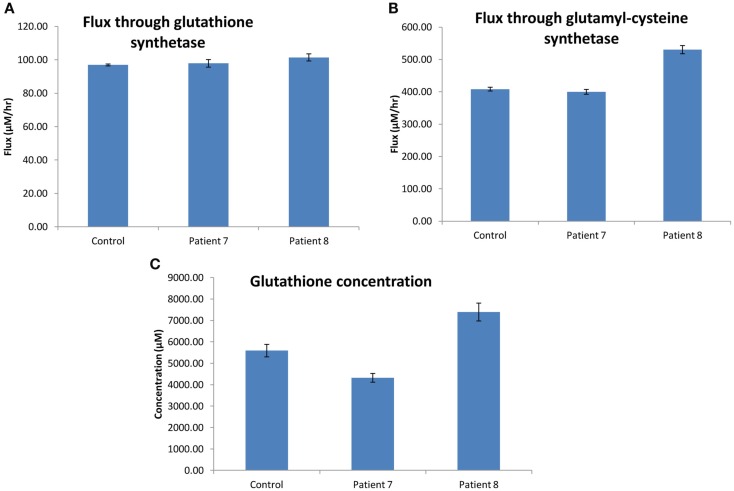
**Glutathione synthesis pathways in dermal fibroblasts from patients with steatosis and control**. **(A)** The average flux through glutathione synthetase (the final step in glutathione synthesis). **(B)** The average flux through glutamylcysteine synthetase (the second to last step in glutathione synthesis). **(C)** The average concentration of glutathione. Error bars are SD.

The flux through glutathione synthetase (final step in glutathione synthesis) was observed to be higher in H0008 than control. The flux through glutathione synthetase was not significantly different between H0007 and control. The flux through glutamylcysteine synthetase (second to last step in glutathione synthesis) was higher in H0008 than in the control. The average concentration of glutathione was higher in H0008 compared to control but lower in H0007 than in control (Figure 8). We then used the model to investigate the reason for this change in glutathione level, i.e., explain the higher glutathione concentration by higher flux through glutathione synthesis pathway. To understand the decrease in glutathione concentration (even though flux through glutathione synthesis seems to be unchanged) we investigated the rate of degradation of glutathione. The increase in expression of glutathione peroxidase led to an increase in the flux through reaction in the model compared to control, which may be a possible explanation for this decrease.

### Correlation between change in flux and change in *V_max_*

One of the assumptions of analyzing expression data was that a change in mRNA expression is correlated to changes in the pathway flux/behavior. The advantage of combining the mRNA expression data to the kinetic model would be to investigate how a change in expression might affect flux (we now assume that a change in mRNA is equal to a change in *V*_max_). To see whether the model predicts different effects on the pathway flux/behavior compared to using the mRNA data alone, we investigated whether there is a correlation between change in the *V*_max_ and change in the flux through the pathway. To test this we investigated two enzymes/reactions which had the largest change in expression; 5-oxoprolinase and DNA (cytosine-5-)-methyltransferase 1 and then one with smaller changes of expression; glutathione synthetase.

**Figure 9:**
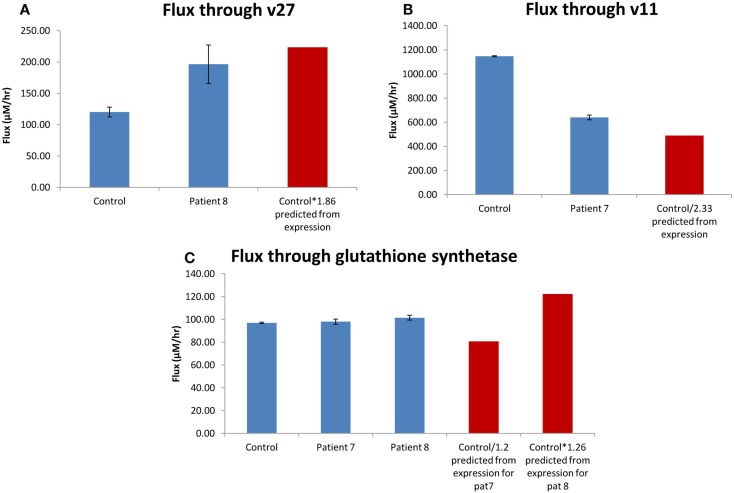
**Correlation between change in flux and change in *V*_max_**. **(A)** The average flux through oxoprolinase (v27) for the patient with steatosis (H0008) and control. **(B)** The average flux through DNA (cytosine-5-)-methyltransferase (v11) for the patient with steatosis (H0007) and control. **(C)** The average flux through glutathione synthetase. The red bar is the predicted value of flux based on mRNA expression. Error bars on model predicted changes in flux are SD.

The expression of 5-oxoprolinase was 1.86 times higher in H0008 than control (H0002). The increase in flux though 5-oxoprolinase (v27 in model; Geenen et al., 2012; corresponding enzyme/reaction) was 1.64 (Figure 9A) implying there is a good correlation between flux and change in *V*_max_. Figure 9A shows that an increase of flux of 1.86 times lies within the error bars of the flux predicted for H0008. DNA (cytosine-5-)-methyltransferase 1 was 2.33 times lower in H0007 than in control. The decrease of flux through v11 was 1.79 times lower implying when compared to 5-oxoprolinase, here the correlation between flux and change in *V*_max_ was not as good (Figure 9B). The glutathione synthetase expression data showed that H0008 had 1.26 times higher expression, and H0007 had 1.2 times lower expression than control. The flux showed that H0008 had a 1.05 times higher flux and H0007 had a 1.01 times higher flux (Figure 9C). This led to a poor correlation between flux and *V*_max_. The insertion of the expression data into a kinetic model of the glutathione pathway allowed us to predict what the effect of expression changes might be on pathway flux. The results suggest that H0008 could have a significant increase in the flux through glutathione synthesis as both gamma-glutamylcysteine synthetase and glutathione synthetase have an increased flux through them. A significant change in flux through glutathione synthesis of H0007 was not observed. Through this model, we have shown that a good correlation between change in flux and change in *V*_max_ may not always be the case. This suggests adopting systems-based approaches (modeling approaches) have an advantage over analyzing mRNA expression data alone to synthesize information.

### Data integration and management

The principle of the livSYSiPS data management platform is to provide a user-friendly interface without superficial elements catering for easy access and exploitation of project data following the model of popular search engines in the World Wide Web. Requirements for the data management system of project livSYSiPS were analyzed taking into account dedicated use cases. Data formats generated in the experiments were assessed to find appropriate metadata models and standard data formats facilitating good representations of experiments in their systems biologic environment.

**Figure 10:**
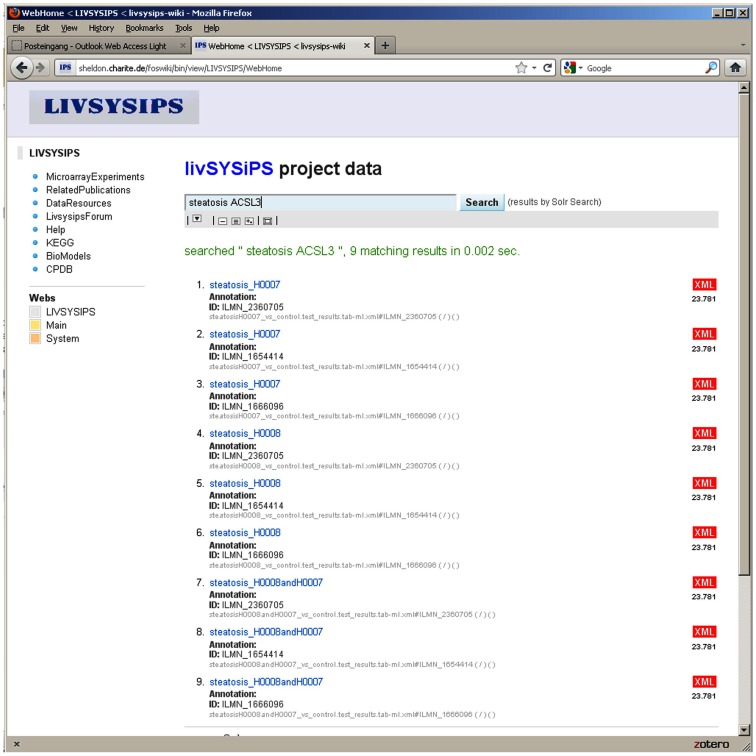
**The user interface of the livSYSiPS data management system provides clear and easy-to-use access to project related data via the central search box and direct links to relevant data categories, e.g., the KEGG link on the left navigation bar addressing pathway analysis results of experimental data**. Shown here are search results for experimental data of steatosis patients focusing on the gene *ACSL3* (long-chain acyl-CoA synthetase) which plays an important role in the fatty acid metabolism pathway. Search results link to follow-up representations and visualizations of models and data.

The Data Integration Platform for Systems Biology Co-operations (DIPSBC; Dreher et al., 2012) which was developed at the Max Planck Institute for Molecular Genetics in Berlin was adapted for the livSYSiPS project at the Charité, Berlin. The DIPSBC is based on wiki technique and the solr search server from the Apache open source project. Wiki and solr platform are brought together via a dedicated solrSearch plugin. The user interface – emphasizing the central search box – is intended to provide clear and easy-to-use access to project data and manifold representations and visualizations of it (Figure 10). In addition to the search engine interface a navigation bar on the front page links directly to the most prominent categories of data. The DIPSBC uses metadata defined via XML descriptions of data. The use of XML as metadata promotes the use of standard formats which are crucial for data sharing. Schema definitions for formats from the Minimum Information for Biological and Biomedical Investigations (MIBBI; Taylor et al., 2008) recommendations have been implemented, e.g., for Minimum Information About a Microarray Experiments (MIAME; Brazma et al., 2001). Systems biology resources, e.g., BioModels (Li et al., 2010a), Kyoto Encyclopedia of genes and genomes (KEGG; Kanehisa et al., 2010), and the ConsensusPathDB (Kamburov et al., 2011b) have been integrated. In a typical application, KEGG pathways enriched in the experimental data can be explored. The system provides statistical parameters for the dedicated tests and a prediction for activation or inhibition via the Bioconductor SPIA package (Tarca et al., 2009). Independent of the significance of the pathway as whole, differentially expressed genes can be visualized in the KEGG pathway chart. The repository allows direct access to raw data and processed data. The livSYSiPS data management is integrated into the system ERASysBio+ SEEK which provides a supervising data management platform for multiple transnational systems biology research projects. The site protected livSYSiPS webpage can be found at the following link: http://sheldon.charite.de/foswiki/bin/view/LIVSYSIPS/WebHome

The site will be available to the general public at the end of the project. For intra-project communication a user forum has been set up which is used for, e.g., exchanging minutes of meetings, slides of talks, etc. and most importantly enables web discussions about project related issues.

The following datasets have so far been incorporated into the database:

- Microarray-based transcriptome data pertaining to the differentiation of healthy human embryonic stem cells and induced pluripotent stem cells into hepatocyte-like cells (Jozefczuk et al., 2011).
- Transcriptome data of fibroblasts derived from patients with steatosis (H0007, H0008) and control (H0002).
- Microarray-based transcriptome data of human hepatocellular xenograft models (Kashofer et al., 2009).
- Reverse Phase Protein Array data.
- Systems biology models from the BioModels database.
- Consensus pathway data integrating multiple pathway databases from ConsensusPathDB.

## Discussion

The current data sets though limited have provided valuable preliminary data pertaining to the etiology of steatosis and have provided the foundation for a more extended analysis based on an extensive collection of liver biopsies, serum, and dermal fibroblast from patients with distinct grades of disease progression.

The transcriptome, proteome, and modeling data obtained from fibroblasts derived from patients with steatosis (H0007, H0008) and control (H0002) has unveiled the following novel results.

The ribosomal protein PRAS40, abundant in steatosis patients has been identified as a crucial regulator of insulin signaling and also as mediator of AKT signals to mTOR (Vander Haar et al., 2007). Furthermore, it has been shown that PRAS40 phosphorylation is decreased under insulin-resistant conditions (Nascimento et al., 2006). Therefore, reduced AKT/mTOR signaling of skin fibroblasts derived from steatosis patients has revealed that the insulin-resistant phenotype is present not only in insulin-metabolizing central organs, e.g., the liver of steatosis patients, but is also manifested in skin fibroblasts derived from these patients.

Our results imply a down-regulation of mTOR under insulin-resistant conditions. However, different correlations between mTOR activity and insulin resistance in animal models (Wang et al., 2010; Medeiros et al., 2011) and humans (Ost et al., 2010; Pereira et al., 2012) is still a matter of an ongoing discussion. In several studies on animals with insulin resistance induced by high-fat diet, activation of mTOR has been observed (Um et al., 2004, 2006; Khamzina et al., 2005).

Network analysis of expression data of steatosis samples vs. control employing tools such as DAVID, ConsensusPathDB, and IMPaLA has revealed several pathways and functional modules of the disease like glutathione metabolism. Based on these pathways and modules a first model prototype of steatosis related processes has been developed employing the modeling and simulation system PyBioS^3^ (Wierling et al., 2007). PyBioS has an integrated interface to the ConsensusPathDB facilitating the semi-automatic development of annotated models integrating signaling pathways, metabolic reactions and gene-regulatory processes.

**Figure 11:**
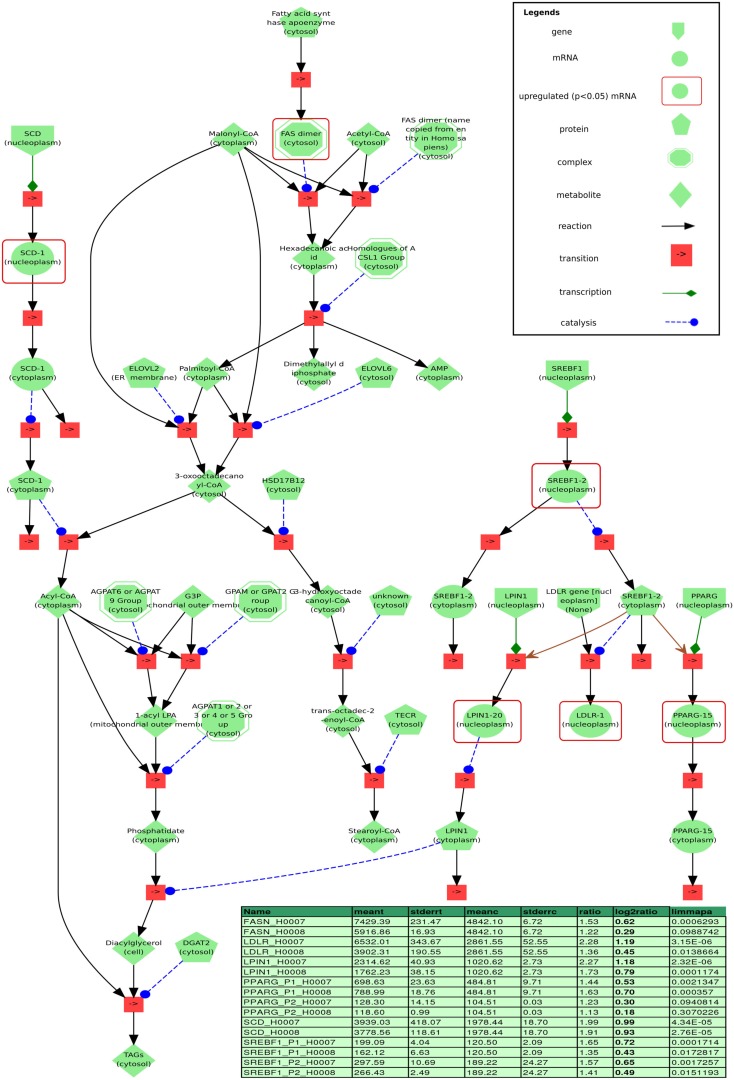
**Visualization of a minimal model of processes found to be related to steatosis based on our data, implemented in the modeling and simulation system PyBioS**. Differentially expressed genes (limmapa < 0.05) of the steatosis patients H0007 and H0008 vs. control H0002 are highlighted by red frames. Two major components of the model that have been identified by expression analysis are the transcription factor sterol regulatory element binding protein 1 (*SREBF1*) and the peroxisome proliferator-activated receptor gamma (*PPARG*). Furthermore, the network integrates important components related to fatty acid synthesis, like fatty acid synthase (*FASN*) and sterol-CoA desaturase (*SCD*), as well as key components of glycerolipid biosynthesis, like *LIPIN1* and *LDLR* (both are regulated by SREBF1). Details of the differentially regulated components are depicted in the table (column headers from left to right: “name”: gene probe name; “meant,” “meanc”: mean treatment values in treatment/steatosis patients and non-steatosis, respectively; “stderrt,” “stderrc”: SE for treatment and control; “ratio,” quotient of meant/meantc; “log2ratio,” log-2 ratio; “limmapa,” *q*-value based on the statistics of the limma-package).

The model prototype which is implemented in PyBioS (see text footnote 3) is based on a minimal network comprising a regulatory network based on the transcription factor SREBF1, linked to a metabolic network of glycerolipid and fatty acid biosynthesis including the downstream transcriptional targets of SREBF1, LIPIN1 (LPIN), and LDLR (Figure 11). The increased expression of *LPIN1* in patients with steatosis, together with the fatty acid-induced translocation of LPIN1 to the endoplasmic reticulum, facilitates increased triacylglycerol synthesis. In addition, Ide et al. (2004) reported a link between SREBF1 and insulin signaling as SREBF1 is shown to directly repress the transcription of IRS-2 and inhibit hepatic insulin signaling.

For subsequent model analysis we will employ a Monte Carlo-based simulation strategy, in which the kinetic parameters are repeatedly sampled from specific probability distributions and used for multiple parallel simulations (Wierling et al., 2012). This facilitates the analysis of large networks of signal transduction, metabolic and gene-regulatory processes, integrating diverse omics data for model refinement, and validation.

We aimed as well to generate a metabolic model of steatotic phenotype. Interestingly among the pathways enriched in steatosis vs. control was the glutathione pathway. Subsequently our investigation involved the mapping of mRNA expression data to a kinetic model of the glutathione synthesis pathway. Adopting this pathway-oriented approach we focused on a subset of complete pathways, rather than all genes of the genome. We have extended this approach to fatty acid biosynthesis, fatty acid metabolism, bile acid pathway, gluconeogenesis, urea cycle, glycolysis, tricarboxylic acid cycle, and glyoxalate shunt; all of which have vital roles in regulation, and are either directly or indirectly involved in the successful functioning of the liver.

Upon data acquisition from all patient-specific samples, various cellular networks will be perturbed by changing environmental conditions of iPS-derived, diseased and healthy cells. The impact of the perturbation will be assessed by constructing key networks from the most significantly dysregulated genes to evaluate any changes as measured and the consequences of these changes as they present themselves throughout the cellular networks.

Ultimately hepatocyte-like cells derived from patient-specific iPS cells will be challenged by free fatty acid overload and high glucose which resemble environmental conditions during the progression of steatosis. Moreover this *in vitro* model will also lay the foundation for identifying drug targets that may be used for treatment of steatosis. Subsequently, hepatocyte-like cells derived from healthy and diseased individuals would allow us to demonstrate the differences in response to clinically relevant drugs and enable to predict hepatotoxicity. We anticipate that the knowledge gained from this minimal number of patient samples will prove advantageous and beneficial to our ongoing analysis with a larger sample of steatosis patients and healthy controls. Undoubtedly, the application of high-throughput transcriptomics, metabolomics proteomic, and modeling to study healthy and stressed liver, will broaden our meager knowledge of steatosis thus, providing deeper insights into the mechanisms underlying the progression to steatohepatitis.

## Conflict of Interest Statement

The authors declare that the research was conducted in the absence of any commercial or financial relationships that could be construed as a potential conflict of interest.

## Footnotes

^1^http://www.ncbi.nlm.nih.gov/geo/

^2^http://www.comp-sys-bio.org/condor-copasi

^3^http://pybios.molgen.mpg.de

## Appendix

**Table A1:** **The range of *V*_max_ inputted into the model for the control model (H0002), patient H0007, and H0008**.

		GSH model values	Patient H0008 low	Patient H0008 high	Patient H0007 low	Patient H0007 high	Control H0002 low	Control H0002 high
*S*-Adenosylhomocysteine hydrolase	Vmfah	1500.00	2005.01	2088.17	1106.75	1236.85	1478.93	1521.07
Betaine-homocysteine methyltransferase	Vmbhmt	24680.00	22348.57	29391.36	22135.64	34694.13	22504.66	26855.34
Cystathionase	Vmcbs	464400.00	783199.77	783199.77	347083.33	456403.00	446706.26	482093.74
4-Nitrophenol-2-monooxygenase	VmP450E1	554.50	488.81	715.53	401.15	899.06	547.34	570.43
DNA-methyltransferase	Vmmeth	1500.00	1127.28	1276.94	607.06	675.93	1422.52	1577.48
Glutamylcysteine synthetase	vGCLf1	2527.00	3590.25	3644.53	2235.68	2371.17	2502.93	2551.07
Gamma-glutamylcyclotransferase	vGCTA	55982.80	78150.56	91180.97	38799.69	55403.46	54293.30	57672.30
Gamma-glutamyltranspeptidase	vGGT	8745.00	8833.35	9624.77	7820.17	9150.53	7634.06	9855.94
Glycine *N*-methyltransferase	Vmmet	597.90	613.03	613.28	548.21	558.17	552.76	643.04
Glutathione peroxidase	vmGPX	188.50	197.74	279.53	153.70	296.32	174.69	202.31
Glutathione reductase	vmgr	28.71	24.19	38.77	23.52	26.78	28.58	28.84
Glutathione synthetase	vGSf1	1548.00	1944.51	1971.09	1252.10	1327.90	1325.19	1770.81
Methionine-adenosyl transferase I	vmMATI	1000.00	937.95	1294.94	978.14	1116.75	989.36	1004.43
Methionine synthase (MS)	vmMS	309.70	333.69	384.73	217.11	233.46	293.39	326.01
5-Oxoprolinase	vop	858301.00	784837.61	1680453.16	749856.81	1318690.96	737257.83	979344.17
